# Identification of Potential Cytokinin Responsive Key Genes in Rice Treated With Trans-Zeatin Through Systems Biology Approach

**DOI:** 10.3389/fgene.2021.780599

**Published:** 2022-02-07

**Authors:** Dwijesh Chandra Mishra, Devender Arora, Neeraj Budhlakoti, Amolkumar U. Solanke, S. V. Amitha CR Mithra, Anuj Kumar, P. S. Pandey, Sudhir Srivastava, Sanjeev Kumar, M. S. Farooqi, S. B. Lal, Anil Rai, K. K. Chaturvedi

**Affiliations:** ^1^ Centre for Agricultural Bioinformatics, ICAR-Indian Agricultural Statistics Research Institute, New Delhi, India; ^2^ National Institute of Animal Science, Rural Development Administration, Jeonju, South Korea; ^3^ ICAR-National Institute for Plant Biotechnology, New Delhi, India; ^4^ Agricultural Education Division, Indian Council of Agricultural Research, New Delhi, India

**Keywords:** WGCNA, systems biology, co-expression, cytokinin, hub genes, QTLs

## Abstract

Rice is an important staple food grain consumed by most of the population around the world. With climate and environmental changes, rice has undergone a tremendous stress state which has impacted crop production and productivity. Plant growth hormones are essential component that controls the overall outcome of the growth and development of the plant. Cytokinin is a hormone that plays an important role in plant immunity and defense systems. *Trans*-zeatin is an active form of cytokinin that can affect plant growth which is mediated by a multi-step two-component phosphorelay system that has different roles in various developmental stages. Systems biology is an approach for pathway analysis to *trans*-zeatin treated rice that could provide a deep understanding of different molecules associated with them. In this study, we have used a weighted gene co-expression network analysis method to identify the functional modules and hub genes involved in the cytokinin pathway. We have identified nine functional modules comprising of different hub genes which contribute to the cytokinin signaling route. The biological significance of these identified hub genes has been tested by applying well-proven statistical techniques to establish the association with the experimentally validated QTLs and annotated by the DAVID server. The establishment of key genes in different pathways has been confirmed. These results will be useful to design new stress-resistant cultivars which can provide sustainable yield in stress-specific conditions.

## Introduction

Rice (*Oryza sativa*) is an important food grain crop that is consumed worldwide (Kubo and Purevdorj, 2004). The human population is estimated to reach approximately 10.7 billion by 2050 (Speidel, 2000) and accordingly, the demand for consumption of rice will also increase. On the other hand, the productivity of rice is not increasing at the same pace due to various reasons such as poor water management, soil depletion, abiotic stresses (e.g., drought, flooding, and salinity), biotic stresses (e.g., damage caused by bacteria, fungi, insects), etc. (Kumar et al., 2017). Therefore, it becomes necessary to understand the underlying mechanisms in crops to sustain production and fulfill the demand of the growing population (Ma and Michailides, 2007). Plants must be able to react rapidly with various abiotic and biotic stress signals and develop efficient defense responses to cope with adverse conditions arising in the field (Kumar et al., 2015). Understanding the crosstalk mechanisms in a stress response could help in the characterization of synergistic and antagonistic mechanisms (Kim et al., 2021). Plant hormones are the key components of these defense and adaptation mechanisms. To facilitate the adaptations to the stresses, various hormonal pathways are upregulated or downregulated. Change in hormonal influence usually affects the degree of resistance or susceptibility to different stresses (O’Brien and Benkova, 2013). Cytokinins (a class of phytohormones) are central regulators of plant growth and development. Cytokinins regulate numerous developmental and physiological processes such as cytokinesis of shoots and roots, reproductive behavior, leaf senescence, and responses to environmental cues, particularly to light and nutrients (Haberer and Kieber, 2002; Werner et al., 2003). Trans-Zeatin (tz) is an active form of cytokinin involved in managing environmental stress. Cytokinin pathway has been widely studied and a huge amount of gene expression data are available in public repositories (Edgar et al., 2002; Leinonen et al., 2011). These data can be better utilized for constructing gene regulatory networks and identifying key genes which will further help in developing improved rice varieties having the ability to produce high yield and resistance to such abiotic stress and adverse conditions (Stuart et al., 2003; Lelandais et al., 2011).

Key genes regulating plant growth and cytokinin responsive genes involved in development process will help in developing better stress tolerant varieties (Zhang et al., 2011, 2012; Li et al., 2019). Co-expression analysis is one of the important ways to construct such a network and identify the most relevant module (Heyer et al., 1999; Hudson et al., 2012; Gaiteri et al., 2014). A statistical approach, Weighted Gene Co-expression Network Analysis (WGCNA) is an effective way to perform such analysis (Tang et al., 2016; Che et al., 2018; Wu et al., 2018) and it has been successfully used previously in identifying important modules and key hub genes related to rice in different conditions (Tan et al., 2017; Zhang et al., 2018). This approach provides an analytical framework for the analysis of microarray and transcriptomic data and helps in relating the gene expression to external traits. Based on various co-expression analysis studies, we found WGCNA as a well-proven approach for the identification of functional modules and co-expressed genes from large biological datasets (Kost et al., 2017; Zhang et al., 2018; Mishra et al., 2021). WGCNA is available as an R package and works on the principle of “guilt by association” (Langfelder and Horvath, 2008), that is, a group of genes are more likely to be associated with each other when they have similar expressions (Gillis and Pavlidis, 2012; van Dam et al., 2018). It uses an unsupervised learning method where large-scale data is clustered by building a tree from bottom to top by connecting the two nearest branches or genes. Modules are formed using the hierarchical clustering method and comprised of genes with similar functions. The modules can be further utilized to identify the important key genes. These key or hub genes are centrally connected genes in different modules which are expected to have an important function and play a critical role in maintaining overall harmony within the cell and development of tissue (Sircar and Parekh, 2015).

In this paper, we have performed co-expression analysis on publicly available microarray data retrieved from the NCBI GEO database (Edgar et al., 2002; Barrett et al., 2013) for cytokinin-responsive genes. We have identified novel key genes in each module using sound statistical approaches of co-expression analysis. Furthermore, we have done the biological validation of the novel key genes using well-established and experimentally validated QTLs (quantitative trait locus) of rice.

## Materials and Methods

### Data Summary

Microarray data related to cytokinin effect on root and leaves of rice were retrieved from the NCBI GEO database with reference series GSE6719 (Hirose et al., 2007). In this experiment, roots and leaves of rice seedlings were treated with *trans*-zeatin (an active form of type-A cytokinin hormone) for 30 and 120 min. Three independent replicate treatments were performed for roots and leaves at each time point. To identify cytokinin responsive genes, dimethyl sulfoxide (DMSO) treated roots and leaves for each time point (30 and 120 min) were used as a control. Three independent replicates of DMSO treatment were performed for each organ per time point. Then, the microarray data were generated by using the Affymetrix GeneChip^®^ rice genome array which contains probes to query approximately 48,564 *japonica* and 1,269 *indica* transcripts. There are 24 samples having accession series (GSE6719). The number of transcripts (features) in the expression data was 57,381, of which annotation data was available for only 23,850 transcripts. We have used only the annotated transcripts for WGCNA-based co-expression analysis.

### Weighted Gene Co-Expression Analysis

To perform the analysis, we first studied the distribution pattern of grown samples using transcript count data and performed principal component analysis. Following, uniform distribution pattern we have used the R package “WGCNA” for constructing the network (Langfelder and Horvath, 2008). The elements in the co-expression matrix are defined as the weighted value of correlation coefficients. Gene co-expression of a pair of genes i and j were calculated using Eq. 1 of an unsigned network:

$$A_{\mathrm{ij}}={\left|\mathrm{cor}\left(E_{i},E_{j}\right)\right|}^{\beta }$$ (1)

where E_i_ and E_j_ consist of expression vector profiles of genes i and j across multiple samples and A_ij_ is the adjacency of the unsigned network. Pearson’s correlation coefficient was used to identify the similarity of genes. The absolute value of correlation is raised to a power *β* to create the adjacency matrix. WGCNA emphasizes high correlation by raising the absolute value of correlation to a power *β* ≥ 1. The correlation network among the genes is built based on the adjacency matrix. Each gene is considered as a node and an edge between two genes is built if it passes a set threshold of co-expression strength. A soft threshold value of 6 was used to determine the significant edges for connecting pairs of genes. Furthermore, modules within the networks were identified by calling the R function “cutreeDynamic” available in the WGCNA package, which helps in identifying the minimum number of large modules with a strong association of genes. The function was applied after identifying the soft threshold of the adjacency matrix, and cut them respectively to get modules related to different functions. These identified modules were further used to identify the key or hub genes responsible for governing specific biological functions in cell development.

In order to identify the modules that were highly related, module similarity was quantified by eigengenes correlation (Shi et al., 2015). The eigengenes of a module are defined as the eigenvector associated with the first principal component of the expression matrix (Langfelder and Horvath, 2007). Highly related modules were identified using module significance. Module significance is defined as the average gene significance of all the genes in the modules to access the association of a module to the phenotype.

### Visualization of Network and Hub Genes Identification

Various methods have been used for the identification of hub genes from a large dataset (Bader and Hogue, 2003; Chin et al., 2014). These methods mainly focused on hub gene identification, based only on gene connection degrees in the gene co-expression network. A heat map was generated to compare the expression pattern across the samples and assigned different colors to genes with similar values (Zhao et al., 2014). Genes are interconnected in each module and possess specific functions. After identifying the most significant module, identification of key or hub genes was carried out using an R package, “dhga” (Differential Hub Gene Analysis) (Das et al., 2017).

### Gene Annotation and Gene Ontology Analysis

The annotation of expressed genes was performed using the Institute for Genomic Research (TIGR) database which includes information regarding biological processes (BP), molecular function (MF), and cellular components (CC) (Ouyang et al., 2007). Furthermore, the identified genes from identified modules were submitted to the OryzaExpress database (Hamada et al., 2011) and converted the ids into probeID (Affymatrix). These ids were subsequently converted into geneid using DAVID (the database for annotation, visualization, and integrated discovery) (Huang et al., 2007) for KEGG (Kyoto Encyclopedia of Genes and Genomes) pathway analysis web server to decipher the role of genes varies from BP, CC, MF, and KEGG pathway analysis respectively against *oryza sativa* database and observed in REVIGO database (Supek et al., 2011). We found the involvement of these hub genes by observing the changes in the expression level of treated and control (normal) conditions [S1_file].

### Validation of Identified Hub Genes

We validated the identified hub genes using *in-silico* approach. In the *in-silico* approach, we identified the chromosome-wise distribution of the hub genes and mapped these genes on chromosomes using web server oryzabase (https://shigen.nig.ac.jp/rice/oryzabase/) (Kurata and Yamazaki, 2006). Further, we used a statistical framework approach to test the association of these hub genes with well-known experimentally validated and genetically rich QTLs data reported for biotic and abiotic stress conditions (Jaiswal et al., 2002). We have used an R package “GSAQ” (https://cran.r-project.org/web/packages/GSAQ) (Das et al., 2018) for mapping the identified hub genes with QTLs on respective chromosomes. GSAQ provides a platform to associate the selected hub genes to the corresponding overlapped QTL-IDs with their genomic positions. Furthermore, the identified hub genes were also validated through pathway analysis.

## Results

### Weighted Co-expression Network Construction and Module Identification

**FIGURE 1:**
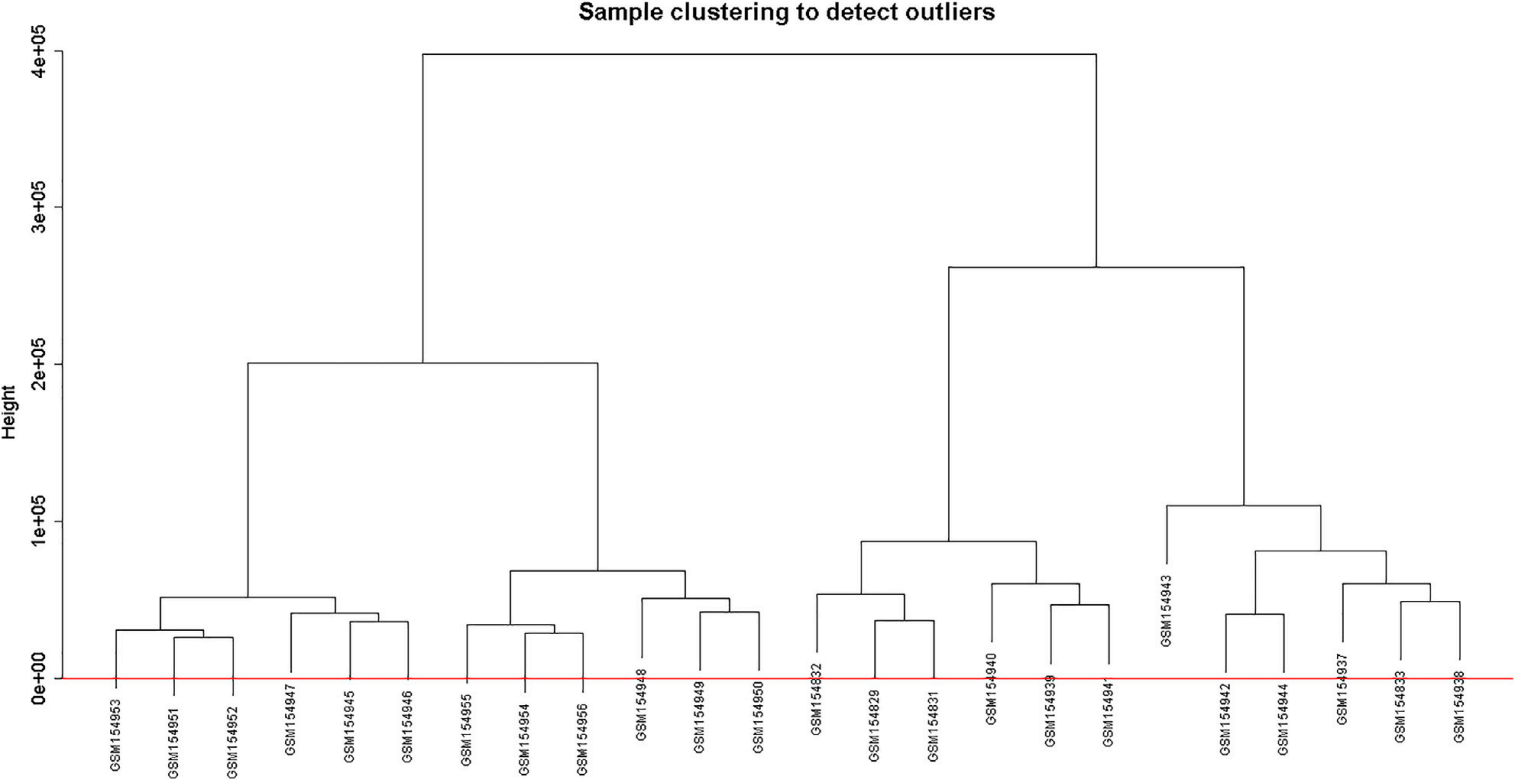
Clustering dendrogram of samples based on their Euclidean distance.

**FIGURE 2:**
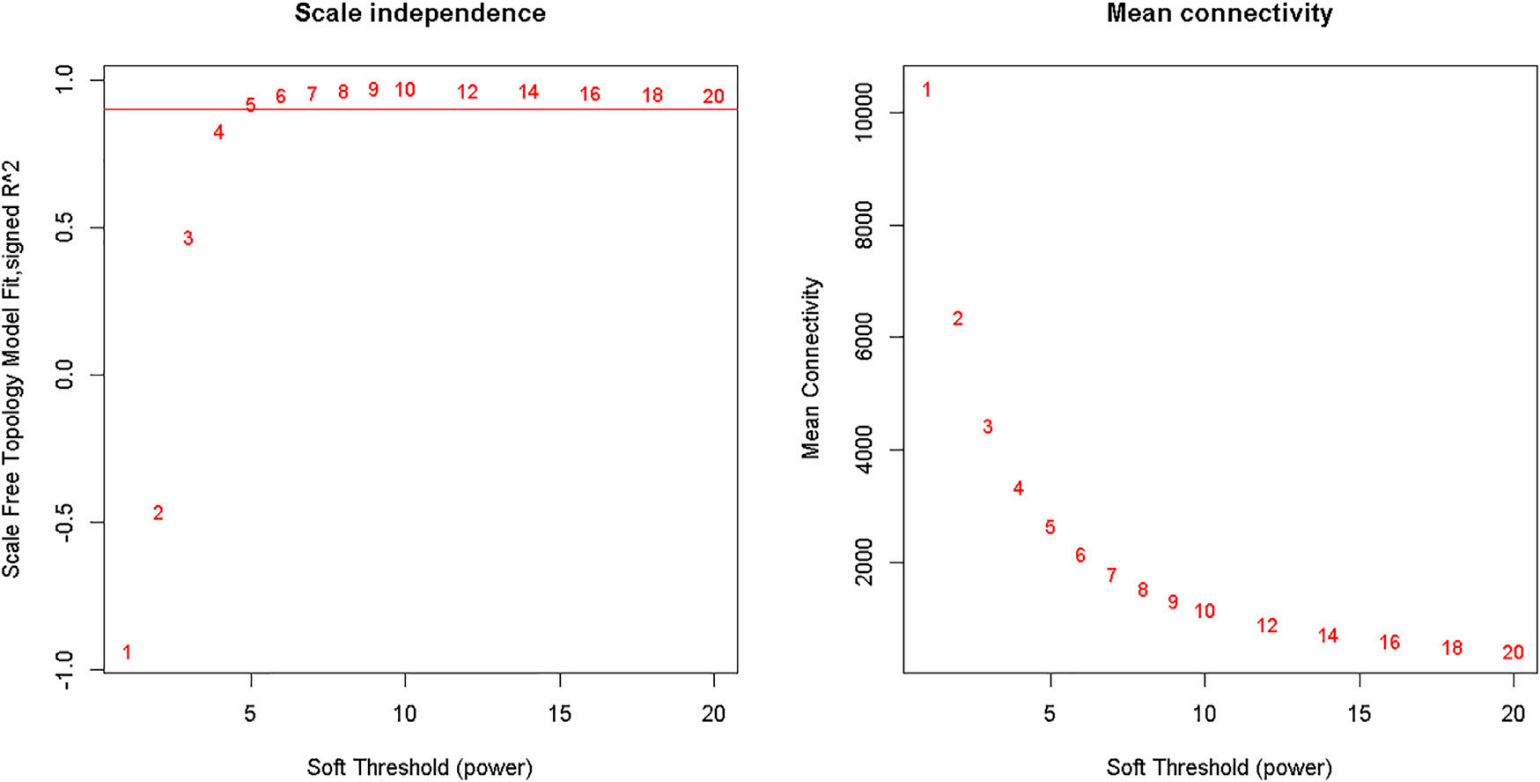
Analysis of network topology for various soft-thresholding powers. The left panel shows the scale-free fit index (*y*-axis) as a function of the soft-thresholding power (*x*-axis). The right panel displays the mean connectivity (degree, *y*-axis) as a function of the soft-thresholding power (*x*-axis).

**FIGURE 3:**
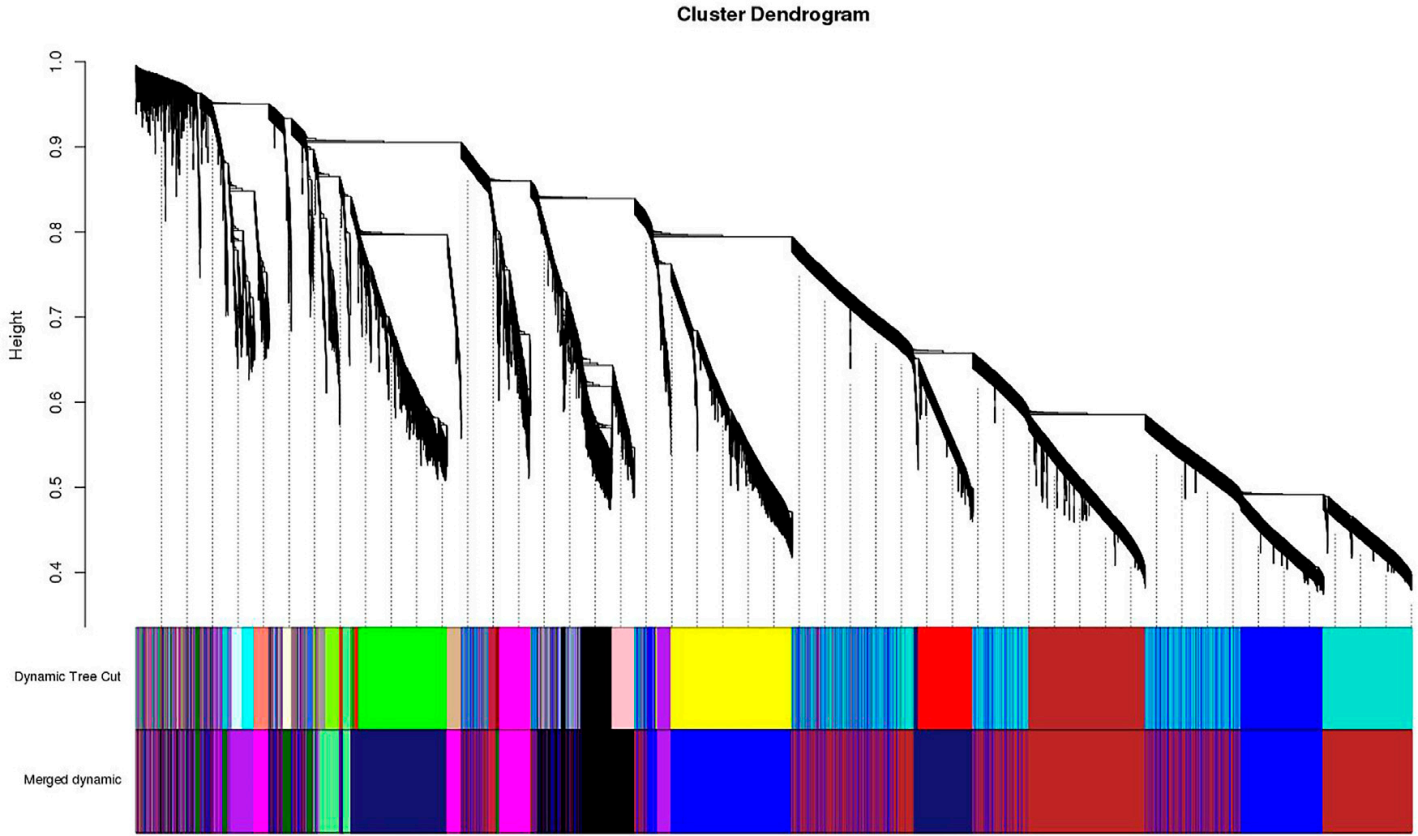
Clustering dendrogram of genes, with dissimilarity based on topological overlap, together with assigned merged module colors and the original module colors.

Clustering of the samples (Figure 1) suggests that there is no outlier present in the data. Power *β* was obtained through two types of graphs given in Figure 2: 1) soft threshold values of *β* (*x*-axis) *vs* scale-free topology model fit scaled *R* ^2^ (*y*-axis) and 2) soft threshold values of *β* (*x*-axis) vs mean connectivity scores (*y*-axis). The optimal value of *β* obtained using these graphs is 6 with R^2^value 0.8. This value of *β* was further used to produce hierarchical clustering (Figure 3).

**FIGURE 4:**
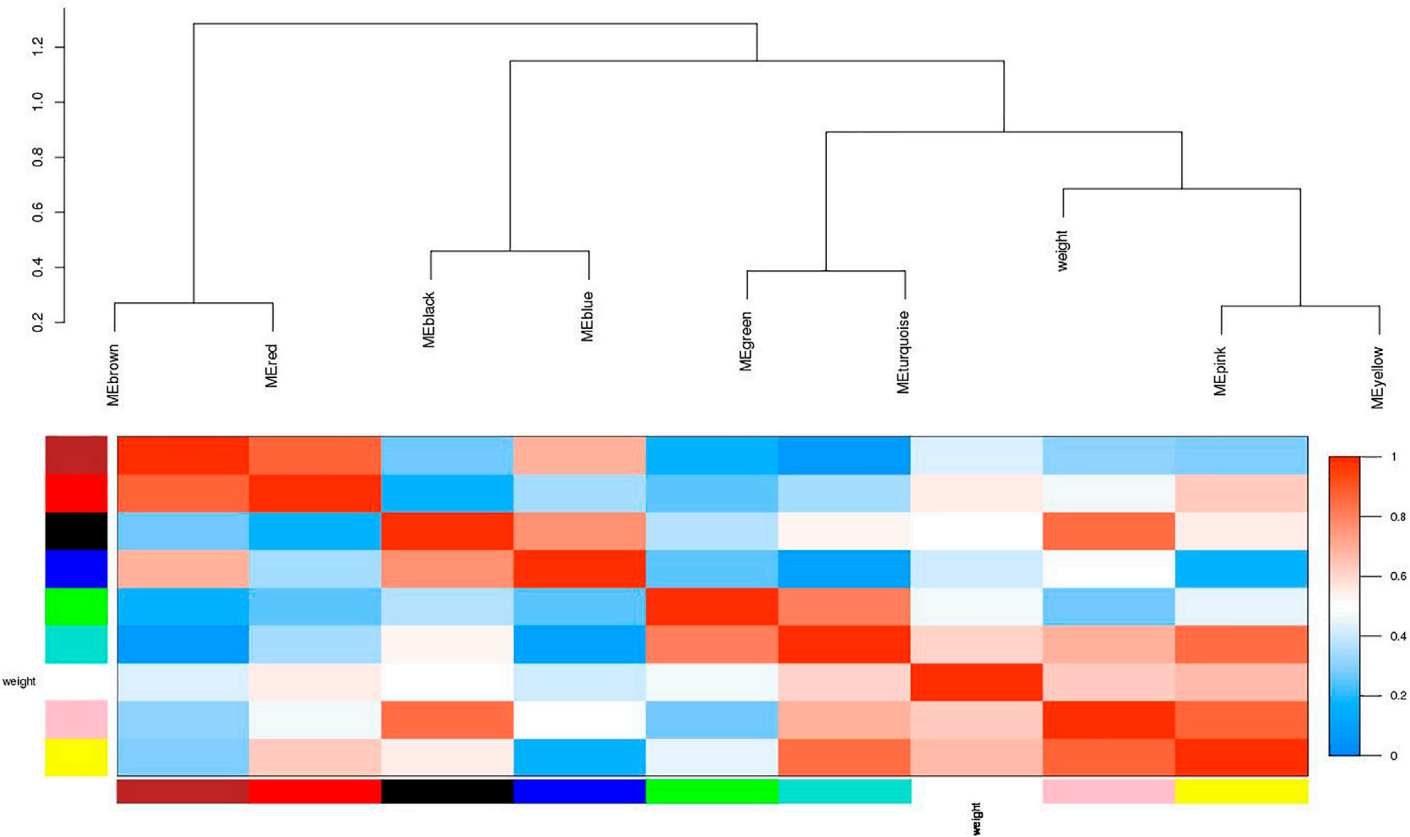
Visualization of the eigengenes network representing the relationships among the modules. The top panel shows a hierarchical clustering dendrogram of the modules based on the dissimilarity score of E_i_ and E_j_ given by 1−cor (E_i_, E_j_). The heatmap in the bottom panel shows the eigengenes adjacency A_ij_= (1 + cor (E_i_, E_j_))/2.

**FIGURE 5:**
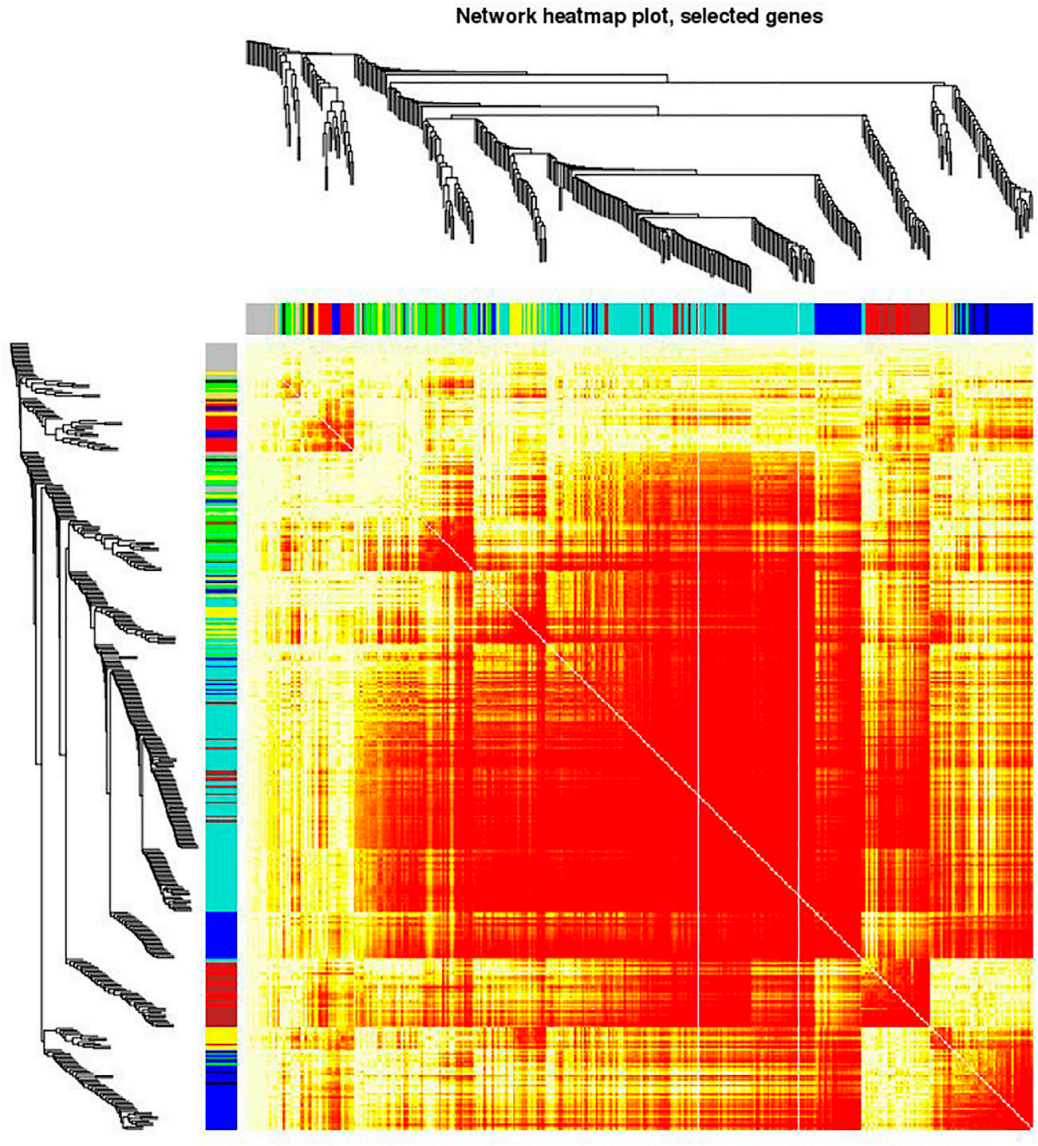
Visualizing the gene network using a heatmap plot. The heatmap depicts the Topological Overlap Matrix (TOM). The light red color represents low overlap and progressively darker shades of red represent higher overlap. Blocks of dark colors along the diagonal depict modules. The gene dendrogram and module assignment are also shown along the left side and the top of the Figure.

A dynamic hierarchical tree algorithm was used to divide the clustering tree constructed from the differentially expressed genes, resulting in 24 different co-expression modules in the data which were named as blue (3,972 genes), brown (3,064 genes), green (1801 genes), yellow (2,387 genes), red (1,272 genes), and turquoise (5,670 genes), Black (1,115 genes), Cyan (275 genes), dark green (85 genes), dark red (113), dark turquoise (83 genes), green yellow (338 genes), grey (35 genes), grey60 (162 genes), light cyan (246 genes), light green (144 genes), light yellow (141 genes), magenta (644 genes), midnight blue (252 genes), pink (835 genes), purple (442 genes), royal blue (122 genes), salmon (296 genes), and tan (326 genes) (Figure 4). After clustering, the genes were grouped into modules (subnetworks) depicted in different colors for easy identification (Figure 5).

**FIGURE 6:**
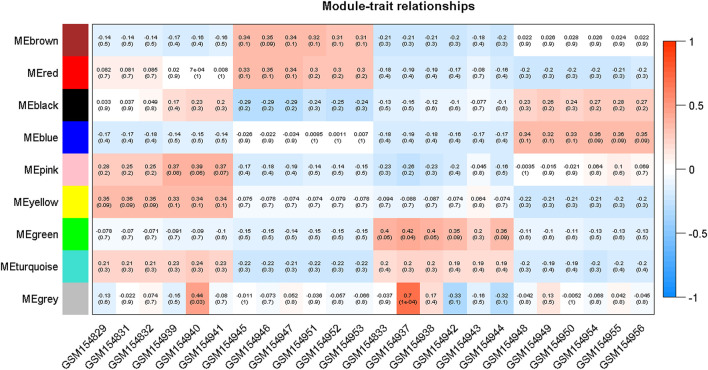
Module-trait associations where each row corresponds to a module or eigengene and a column to a trait. Each cell contains the corresponding correlation and *p*-value. The table is color-coded by correlation according to the color legend mentioned in the Figure.

Moreover, in order to explore the relationship between identified modules and the experimental samples (traits), we calculated and tested the correlation coefficients. These correlation coefficients along with their *p*-values for module-trait relationship were depicted (Figure 6). In Figure 6, the red color shows a strong positive correlation and the blue color displays a strong negative correlation. Furthermore, the centralized hub genes were identified from these modules through statistical analysis with the help of R-package “dhga” using a weighted gene score. A total of 36 hub genes were identified in 9 modules and their detailed description (function/annotation, location, and accession number) are mentioned in Table 1; Table 2; Table 3; Table 4; Table 5; Table 6: Table 7; Table 8; Table 9.

**TABLE 1:** Identified hub genes with respect to their level of connectivity in the turquoise module.

S.No	Hub gene	Function/Annotation	Locus location	Acc. No
1	Os04g0295400	Horcolin/Jasmonate-induced protein, putative, expressed	LOC4335407/Chr 4, NC_029259.1 (12989808.12993215)	AK067477
2	Os03g0425800	uncharacterized LOC4333157	LOC4333157/Chr 3, NC_029258.1 (17780383.17784105)	AK100427
3	Os02g0731200	MADS-box transcription factor 57	LOC4330621/Chr 2, NC_029257.1 (30456659.30462758)	AY177702
4	Os01g0558800	uncharacterized LOC4324676	LOC4324676/Chr 1, NC_029256.1 (21151568.21155639, complement)	AK068120

**TABLE 2:** Identified hub genes with respect to their level of connectivity in the blue module.

S.No	Hub gene	Function	Locus location	Acc. No
1	Os07g0424400	probable cellulose synthase A catalytic subunit 3 [UDP-forming]	LOC4343049/Chr 7, NC_029262.1 (13741571.13747205, complement)	AK120236
2	Os02g0511800	uncharacterized LOC4329457	LOC4329457/Chr 2, NC_029257.1 (18343563.18347153, complement)	AK069817
3	Os08g0505200	uncharacterized LOC4345975	LOC4345975/Chr 8, NC_029263.1 (24986078.24990484)	AK067190
4	Os01g0276800	Glucosidase 2 subunit beta	LOC4324264/Chr 1, NC_029256.1 (9702711.9709700, complement)	AK108476

**TABLE 3:** Identified hub genes with respect to their level of connectivity in the brown module.

S.No	Hub gene	Function	Locus location	Acc. No
1	Os01g0743600	ATP-dependent protease La domain containing protein, expressed	LOC4326165/Chr 1, NC_029256.1 (31078612.31088072, complement)	AK102317
2	Os06g0319800	Os01g0512300	Os01g0512300/Chr 1, NC_008394.3 (18047906.18050788, complement)	AK107048
3	Os07g0636600	dirigent protein 5	LOC4344033/Chr 7, NC_029262.1 (26445635.26446517, complement)	AK106022
4	Os05g0128100	uncharacterized LOC4337691	LOC4337691/Chr 5, NC_029260.1 (1652011.1653860)	AK108556

**TABLE 4:** Identified hub genes with respect to their level of connectivity in the green module.

S.No	Hub gene	Function	Locus location	Acc. No
1	Os07g0171300	uncharacterized LOC4342515	LOC4342515/Chr 7, NC_029262.1 (3773297.3779772)	AK100663
2	Os03g0610800	serpin-ZXB	LOC4333434/Chr 3, NC_029258.1 (23054420.23068346)	AK107194
3	Os01g0827600	exocyst complex component EXO70B1	LOC4327525/Chr 1, NC_029256.1 (35412698.35416714)	AK122173
4	Os08g0200400	KH domain-containing protein At4g18375	LOC4344900/Chr 8, NC_029263.1 (5810049.5816102, complement)	AK067859

**TABLE 5:** Identified hub genes with respect to their level of connectivity in the yellow module.

S.No	Hub gene	Function	Locus location	Acc. No
1	Os09g0442400	protein GAMETE EXPRESSED 1	LOC4347181/Chr 9, NC_029264.1 (16443693.16448158)	AK106970
2	Os08g0522400	putative l-ascorbate peroxidase 6	LOC4346078/Chr 8, NC_029263.1 (2,5971775.25974968, complement)	AK065893
3	Os03g0654600	chlorophyll (ide) b reductase NOL, chloroplastic	LOC4333604/Chr 3, NC_029258.1 (25520290.25525342, complement)	CB669633
4	Os07g0550600	benzyl alcohol O-benzoyltransferase	LOC4343545/Chr 7, NC_029262.1 (21854513.21856895)	AK109553

**TABLE 6:** Identified hub genes with respect to their level of connectivity in the red module.

S.No	Hub gene	Function	Locus location	Acc. No
1	Os12g0566000	boron transporter 1	LOC4352546/Chr 12, NC_029267.1 (23248819.23253256)	AK100510
2	Os04g0658300	ribulose bisphosphate carboxylase/oxygenase activase, chloroplastic	LOC4337267/Chr 4, NC_029259.1 (33575149.33579656, complement)	AK067399
3	Os05g0358200	DNA primase small subunit	LOC9267485/Chr 5, NC_029260.1 (17006498.17011733, complement)	AK073973
4	Os11g0707000	ribulose bisphosphate carboxylase/oxygenase activase, chloroplastic	LOC4351224/Chr 11, NC_029266.1 (28932976.28936094, complement)	CB673145

**TABLE 7:** Identified hub genes with respect to their level of connectivity in the black module.

S.No	Hub gene	Function	Locus location	Acc. No
1	Os11g0491400	uncharacterized LOC4350546	LOC4350546/Chr 11, NC_029266.1 (17379762.17382920)	AK068341
2	Os07g0271500	bisdemethoxycurcumin synthase	LOC4342896/Chr 7, NC_029262.1 (10018732.10020733)	AK109558
3	Os09g0482740	uncharacterized LOC9271634	LOC9271634/Chr 9, NC_029264.1 (18576964.18581257)	AK061852
4	LOC4338611	lichenase-2	LOC4338611/Chr 5, NC_029260.1 (18106236.18110996, complement)	CB628871

**TABLE 8:** Identified hub genes with respect to their level of connectivity in the pink module.

S.No	Hub gene	Function	Locus location	Acc. No
1	Os07g0187700	SEC12-like protein 1	LOC4342601/Chr 7, NC_029262.1 (4682485.4687647)	AK111777
2	Os05g0568800	bradykinin-potentiating and C-type natriuretic peptides	LOC4339650/Chr 5, NC_029260.1 (28310927.28311938)	AK059883
3	Os04g0334700	aspartic proteinase-like protein 2	LOC4335504/Chr 4, NC_029259.1 (15623634.15653865, complement)	AK120870
4	Os03g0359000	uncharacterized LOC4332880	LOC4332880/Chr 3, NC_029258.1 (13927427.13935150, complement)	AK064512

**TABLE 9:** Identified hub genes with respect to their level of connectivity in the grey module.

S.No	Hub gene	Function	Locus location	Acc. No
1	Os07g0131600	hexose carrier protein HEX6	LOC4342334/Chr 7, NC_029262.1 (1669273.1671384)	AK068296
2	Os03g0704100	probable plastid-lipid-associated protein 4, chloroplastic	LOC4333849/Chr 3, NC_029258.1 (28304914.28307925)	AK070474
3	Os06g0130400	probable aminotransferase ACS12	LOC4340002/Chr 6, NC_029261.1 (1629690.1633597)	AK065212
4	Os03g0322500	14 kDa zinc-binding protein	LOC4332685/Chr 3, NC_029258.1 (11672780.11677395)	AK121029

### Validation of Identified Hub Genes

**FIGURE 7:**
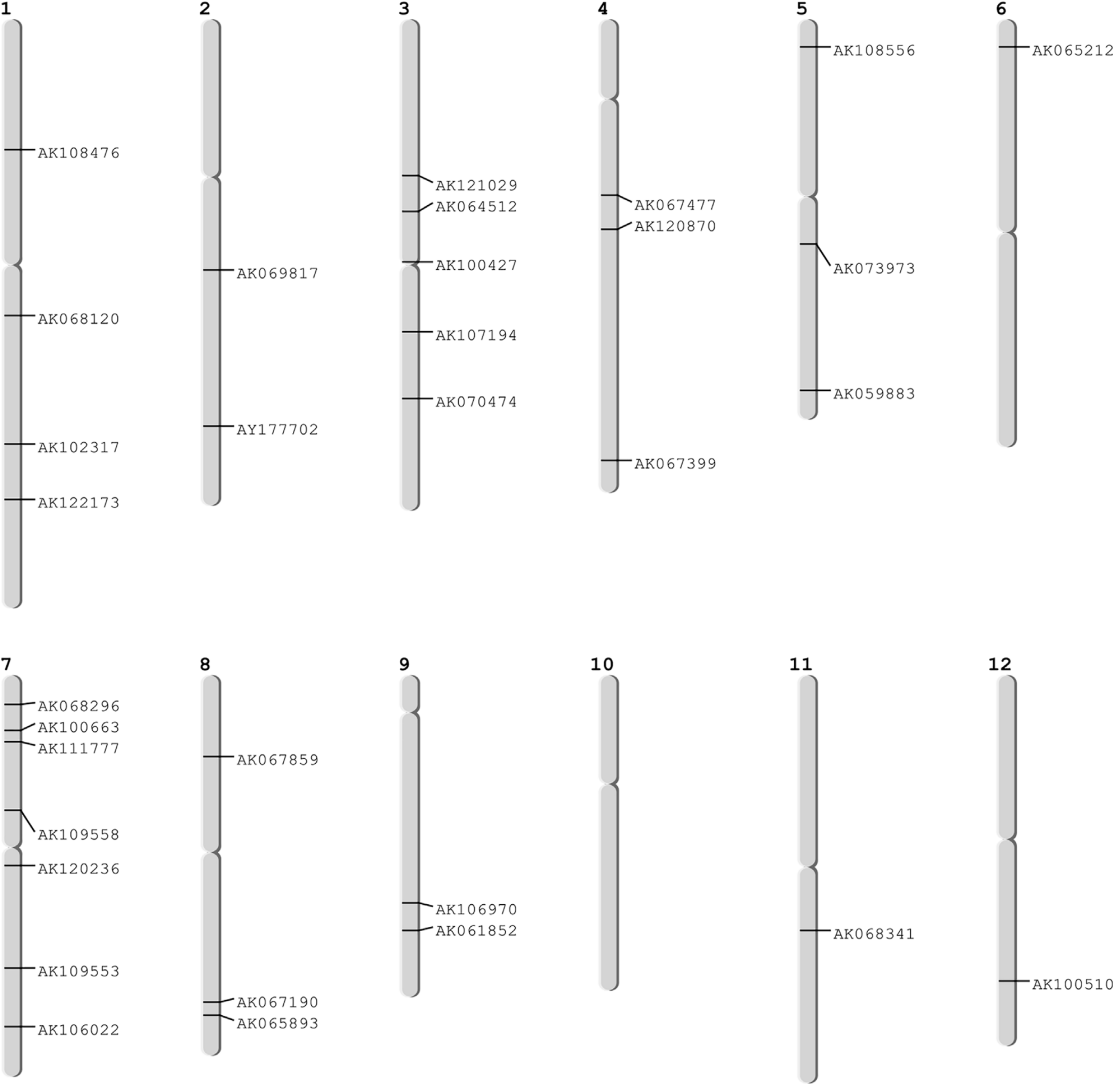
Mapped hub genes on different chromosomes.

**FIGURE 8:**
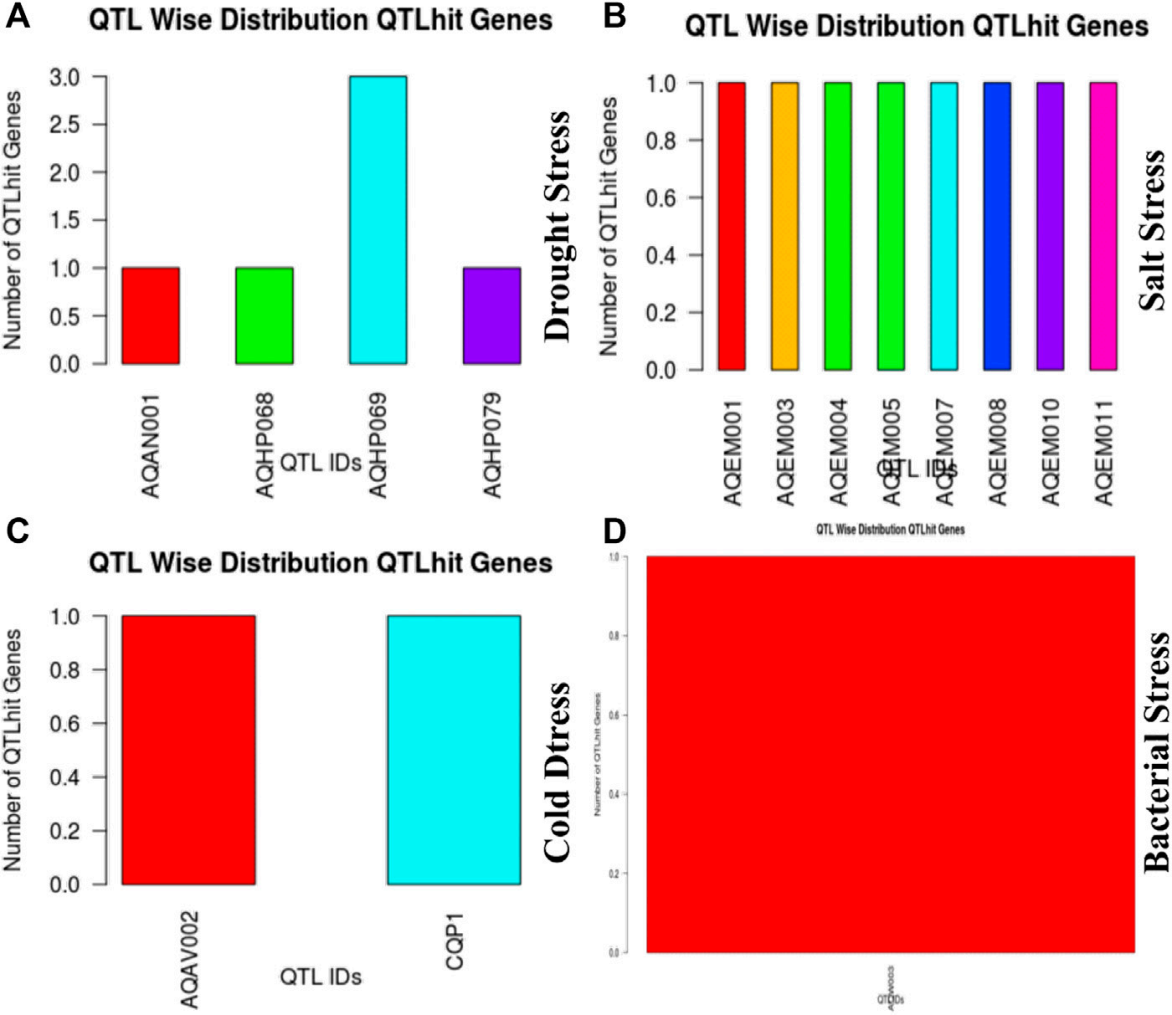
Hub gene distribution on chromosome to the corresponding QTL ids.

**FIGURE 9:**
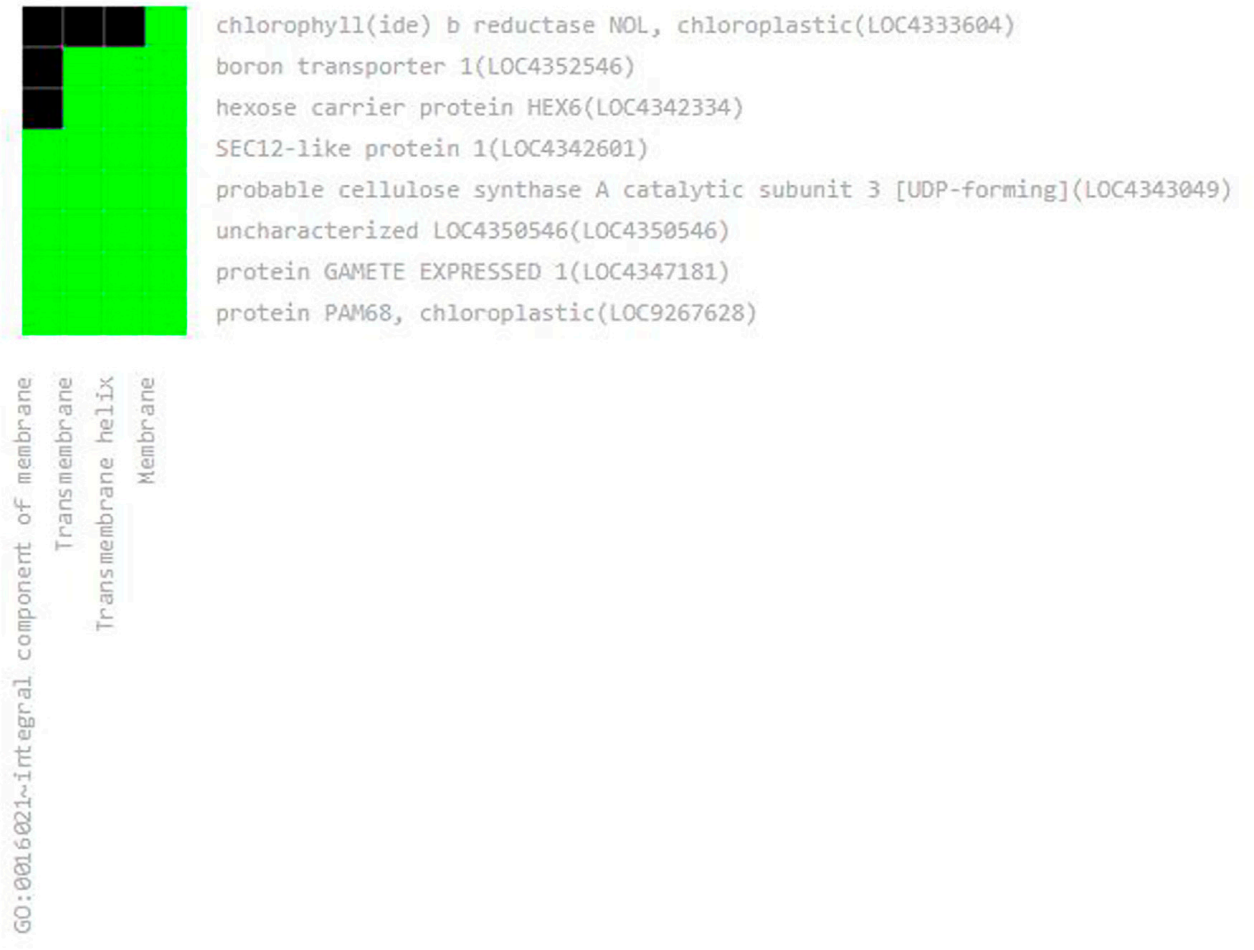
The classification of identified hub genes represented by heat map using DAVID software. It is noted that eight genes (*y*-axis) are classified into four classes (*x*-axis).

These 36 identified hub genes were mapped on rice chromosomes and 32 genes were found to be located at various rice chromosomes (Figure 7). In the process of finding the association of these genes with the well-known QTLs related to salt, cold, drought, bacterial stress, we found that 17 out of the 36 identified hub genes were associated with these QTLs mapped on various chromosomes (Figure 8). We also performed pathway analysis and found that the expression of these genes either increased or decreased during the period of treatment in five different pathways. Heat map analysis was conducted through DAVID to produce a matrix of enriched GO terms with the identified genes. The green and black color shading on the heat map matrix indicate a positive and negative correlation between the enriched GO term and identified DMSO and tz-treated hub genes, respectively (Figure 9).

## Discussion

**FIGURE 10:**
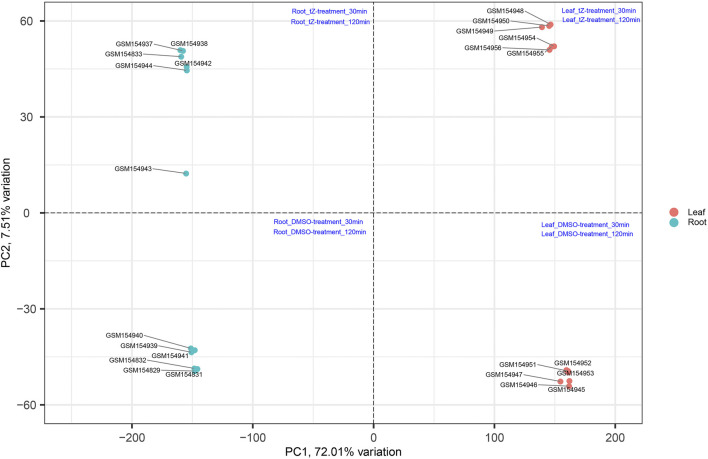
Distribution of samples based on different stress at different time intervals and obtained uniform distribution of data in all four coordinates (Szklarczyk et al., 2017).

The productivity of rice is severely affected due to various biotic and abiotic stresses. Therefore, to develop a variety that is resistant to these stresses, there is an urgent need to identify important hub genes governing the whole production process. Rice genome consists of around 58,000 genes (Cao et al., 2012) and conventional approaches can identify several hundred genes related to these stresses. However, by using conventional approaches, it becomes difficult to identify the few centrally important genes that play important roles in cellular functions to cope with these stresses. This problem can be solved by applying systems biology approaches (Arora et al., 2019). In this study, we have performed a comprehensive analysis on existing data retrieved from NCBI to understand the potential genes and mechanisms involved in such processes by first performing PCA to validate the distribution and uniformity of data evenly (Figure 10) and subsequently obtained significant modules associated with the biological functions regulating the growth and development of the plant. Moreover, hub genes in these modules were also identified that play an important role during cytokinin signaling and are crucial in plant growth and development.

Amongst the identified modules, the hub genes identified in these modules were observed to be involved in various processes. For example, in the turquoise module, the top hub gene (Os04g0295400) is located in chr4 and its important function is to encode Jasmonate-induced protein. Though little is known about this function in rice, Jasmonate-induced proteins are already reported for immunity and development in other plants (Wasternack and Hause, 2013; Campos et al., 2014). In-depth characterization of this gene is further required as this family of genes are reported to play important roles such as in the defense systems against lethal disease and bacterial blight (Yamada et al., 2012; Taniguchi et al., 2014) and it may also be involved in stress management as both these stresses (abiotic and biotic stress) are interlinked with each other (Cao et al., 2017). Similarly, in the blue module, we identified the Os07g0424400 hub gene in chr7 that played an important role in cellulose synthase A catalytic subunit 3 [UDP-forming] that governs a major mechanism of cell wall formation (Taylor et al., 2000) and ultimately helps in supporting the plant growth against abiotic stress. In the brown module, the top potential hub gene, Os01g0743600, is located on chr1 with the reported function of ATP-dependent protease La domain containing protein (Koodathingal et al., 2009) and it is one of the key components in providing protection against the harmful effects of unfolded proteins. It is activated by stress conditions in the endoplasmic reticulum (ER) and it supports plant defense as well as response to abiotic stresses (Bao and Howell, 2017).

The top potential key gene in the green module*,* Os07g0171300, is still not fully characterized, but annotation results suggest it has a key influence in the protein kinase-like domain superfamily which is believed to be a conserved protein domain mainly involved in most signaling and regulatory processes in the eukaryotic cell, and as a switch in controlling biological processes such as metabolism, transcription, cell moment, apoptosis, etc.

In the yellow module, the identified key gene Os12g0566000 codes for boron transporter 1, and is located at LOC4352546 of Chr12 and mapped with QTL identification number AK100510. Boron is essential for maintaining the integrity of plants cell walls. It exhibits an important structural role in shaping the cell by providing mechanical strength via cross-linking of cell wall rhamnogalacturonan II (RG-II) to form a stable three-dimensional pectic network which contributes to the mechanical properties of cell wall structure (Funakawa and Miwa, 2015). It is also reported that boron expression deficiency inhibits plant photosynthetic capacity (Zhao and Oosterhuis, 2002; Kastori et al., 2008) and directly impacts the total yield of the crop.

In the red module, the identified key gene Os09g0442400 codes for protein Gamete expressed 1, and is located at LOC4347181 of Chr9 and mapped with QTL identification number AK106970. Gamete expressed 1 protein is mainly responsible for fertilization. It has a dual function during gametophyte development and early embryogenesis and it is required for correct pollen maturation (Engel et al., 2005). In the pink module, the identified key gene Os07g0187700 codes for protein SEC12-like protein1, and is located at LOC4342601 of Chr7 and mapped with QTL identification number AK111777. Phosphate Transporter 1 (PHT1) is a plant-specific SEC12 gene that encodes phosphate transporter involved in phosphate uptake by facilitating the trafficking of PHT1-1/PHT1; 1 from the ER to the plasma membrane that enables the ER exit of a high-affinity phosphate transporter (González et al., 2005). The top key hub gene identified in the grey module, Os07g0131600, codes for HEX6 protein which is one of the hexose carrier proteins, and is located at LOC4342334 of Chr7 mapped with QTL identification number AK068296. HEX6 protein has an active uptake of hexose with an important role in glucose/hydrogen symport (Boles and Hollenberg, 1997). These different hub genes directly or indirectly govern the main function of the positive build-up of overall cell growth but there are leftover top key genes Os07g0171300 in the green module and Os11g0491400 in the black modules that need to be further characterized to understand their role at different stages of crop development.

**FIGURE 11:**
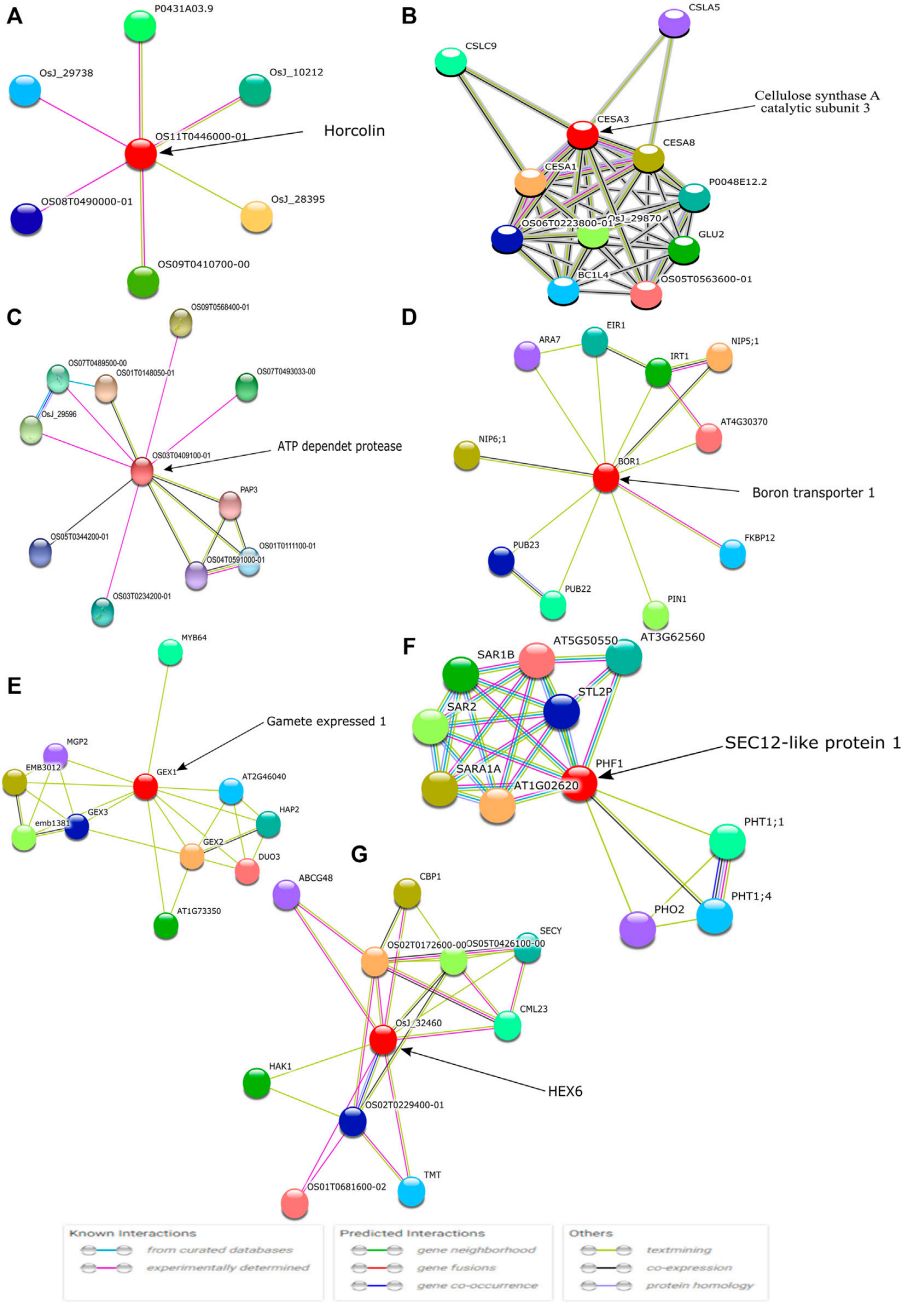
The identified top hub genes are denoted with circles and are known as the first shell of interactors. Each color symbol signifies a specific interaction either known or predicted, as mentioned in previous studies.

Further, the identified key hub genes were visualized using the STRING database for protein-protein interaction (Szklarczyk et al., 2017). The STRING database helped in the identification of direct (physical) interactions and indirect (functional) interactions as long as the interactions were specific and biologically meaningful. Out of the 9 obtained modules, seven genes were found to be associated with neighboring proteins (Figure 11).

**FIGURE 12:**
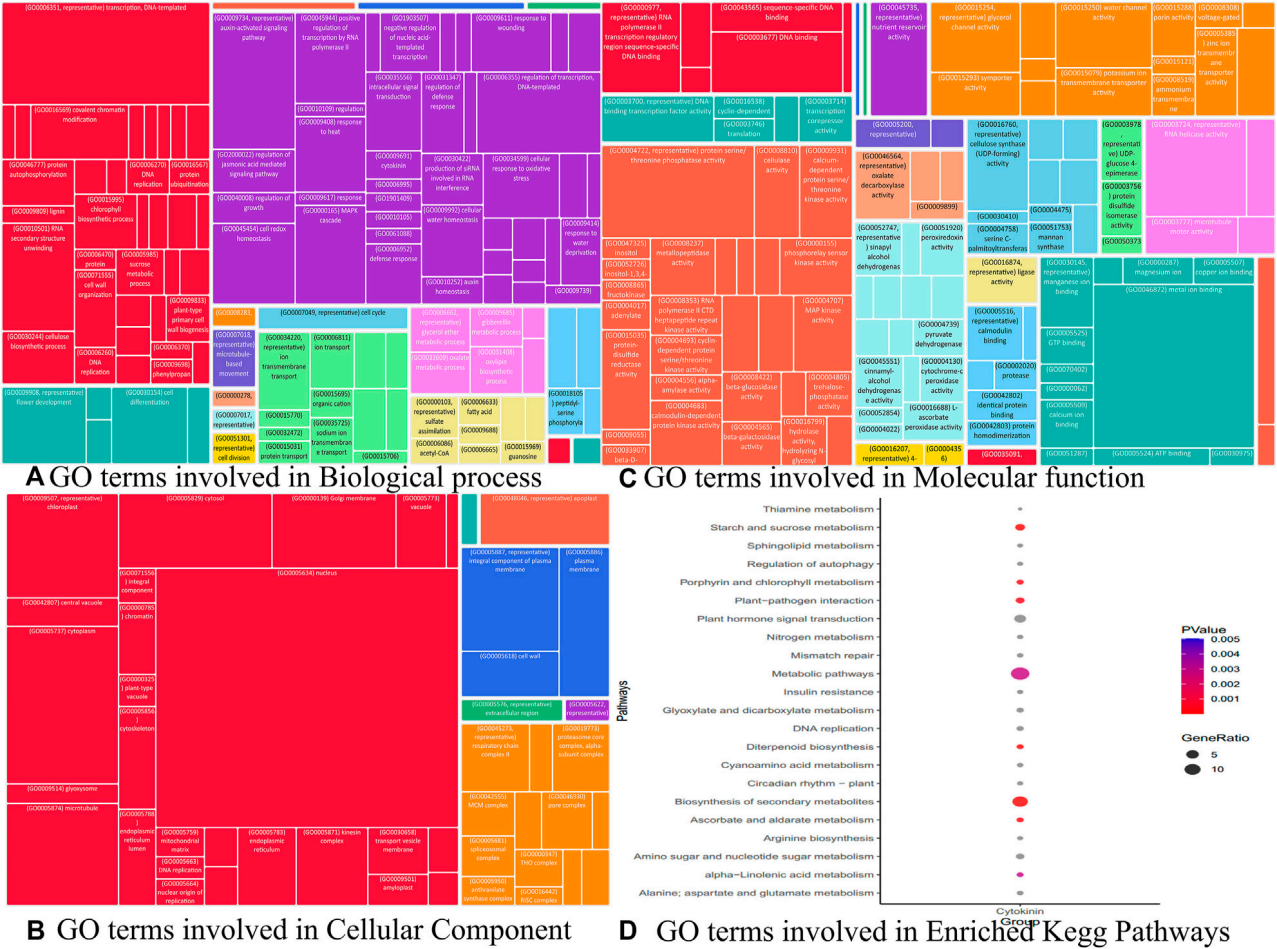
Gene ontology analysis viz biological process, cellular component, and molecular function terms associated with different modules performed using REVIGO.

Similarly, we identified other important hub genes in each module (Table 1 to Table 9) which are not yet fully explored with respect to cytokinin signaling to maintain the harmony of cell and rice growth mechanisms. Although we identified 36 key genes, we were interested in understanding the role of genes in various processes such as BP, CC, and MF to further delineate the role of these genes in playing different roles in the development of cytokinin-related responses. The Gene Ontology terms in these processes include transcription (GO:0006351), auxin-activated signaling pathway (GO:0009734), MAPK cascade (GO:0000165), and regulation of transcription in BP (GO:0006355) (Figure 12A), whereas the GO terms mostly constituted in CC include nucleus (GO:0005634), cytoplasm (GO:0005737), chloroplast (GO:0009507), and microtubule (GO:0005874) (Figure 12B) and composed of molecular functions metal ion binding (GO:0046872), protein serine/threonine phosphatase activity (GO:0004722), ATP-dependent RNA helicase activity (GO:0004004), and RNA polymerase II regulatory region sequence-specific DNA binding (GO:0000977) are some (Figure 12C). Likewise, the Kegg pathway also revealed the enrichment of GO terms such as plant hormone signal transduction (osa04075), starch and sucrose metabolism (osa00500), and diterpenoid biosynthesis (osa00904) (Figure 12D). In the absence of wet lab experiments, these identified hub genes were validated with the help of well-known QTLs and pathways using an *in-silico* approach. The analysis indicates the involvement of identified hub genes in these stress conditions as these genes are found to be associated with biotic and abiotic stress-related QTLs and pathways. These results indicate the role of identified hub genes in the regulation of plant growth and development. However, these hub genes need further attention at the molecular level through wet lab experiments to improve the traits which will be useful in enhancing the productivity of the crop.

## Data Availability

The original contributions presented in the study are included in the article/Supplementary Material, further inquiries can be directed to the corresponding author.

## Abbreviations

QTL, Quantitative trait locus; WGCNA, Weighted correlation network analysis; GEO, Gene expression omnibus; tz, trans-zeatin; DMSO, Dimethyl sulfoxide; dhga: Differential hub gene analysis; GSAQ, Gene Set Analysis with QTL; ER, Endoplasmic reticulum; KEGG, Kyoto Encyclopedia of Genes and Genomes; DAVID, Database for annotation, visualization, and integrated discovery; TOM, Topological overlap matrix; GO, Gene ontology; PHT1, Phosphate transporter 1.
