# Supplementary material for: Identification of Potential Cytokinin Responsive Key Genes in Rice Treated With Trans-Zeatin Through Systems Biology Approach
Source: Front Genet. 2022 Feb 7;12:780599. doi: 10.3389/fgene.2021.780599 (PMC8859635; doi:10.3389/fgene.2021.780599)
Supplement: Supplementary file 1 [file Table1.DOCX]

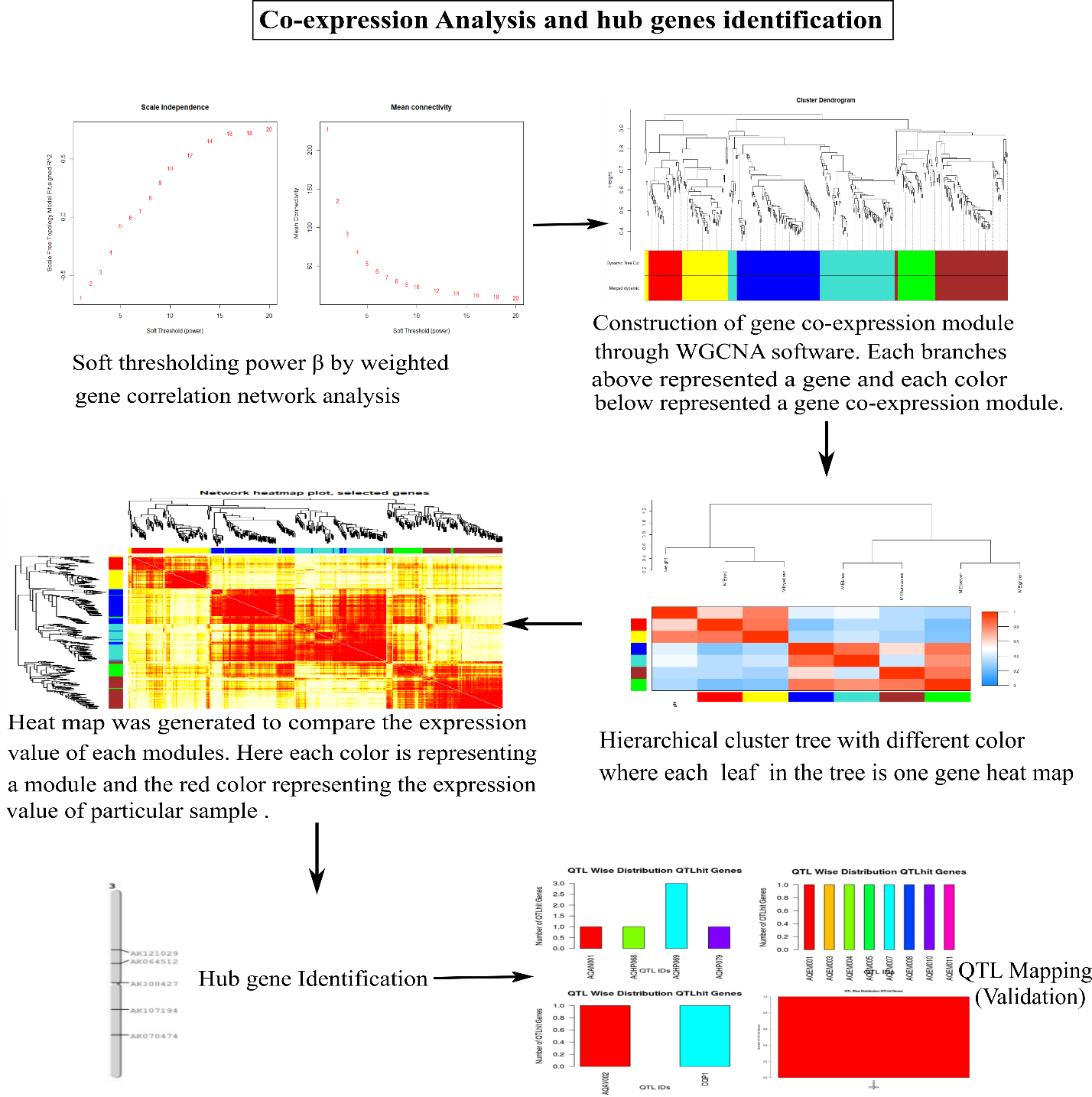


**Graphical Abstract**: The step by step procedure for identification of different hub genes and validation by mapping with QTL-Ids of different stress related genes.
