# Supplementary material for: Identification of Potential Cytokinin Responsive Key Genes in Rice Treated With Trans-Zeatin Through Systems Biology Approach
Source: Front Genet. 2022 Feb 7;12:780599. doi: 10.3389/fgene.2021.780599 (PMC8859635; doi:10.3389/fgene.2021.780599)
Supplement: Supplementary file 2 [file DataSheet2.docx]

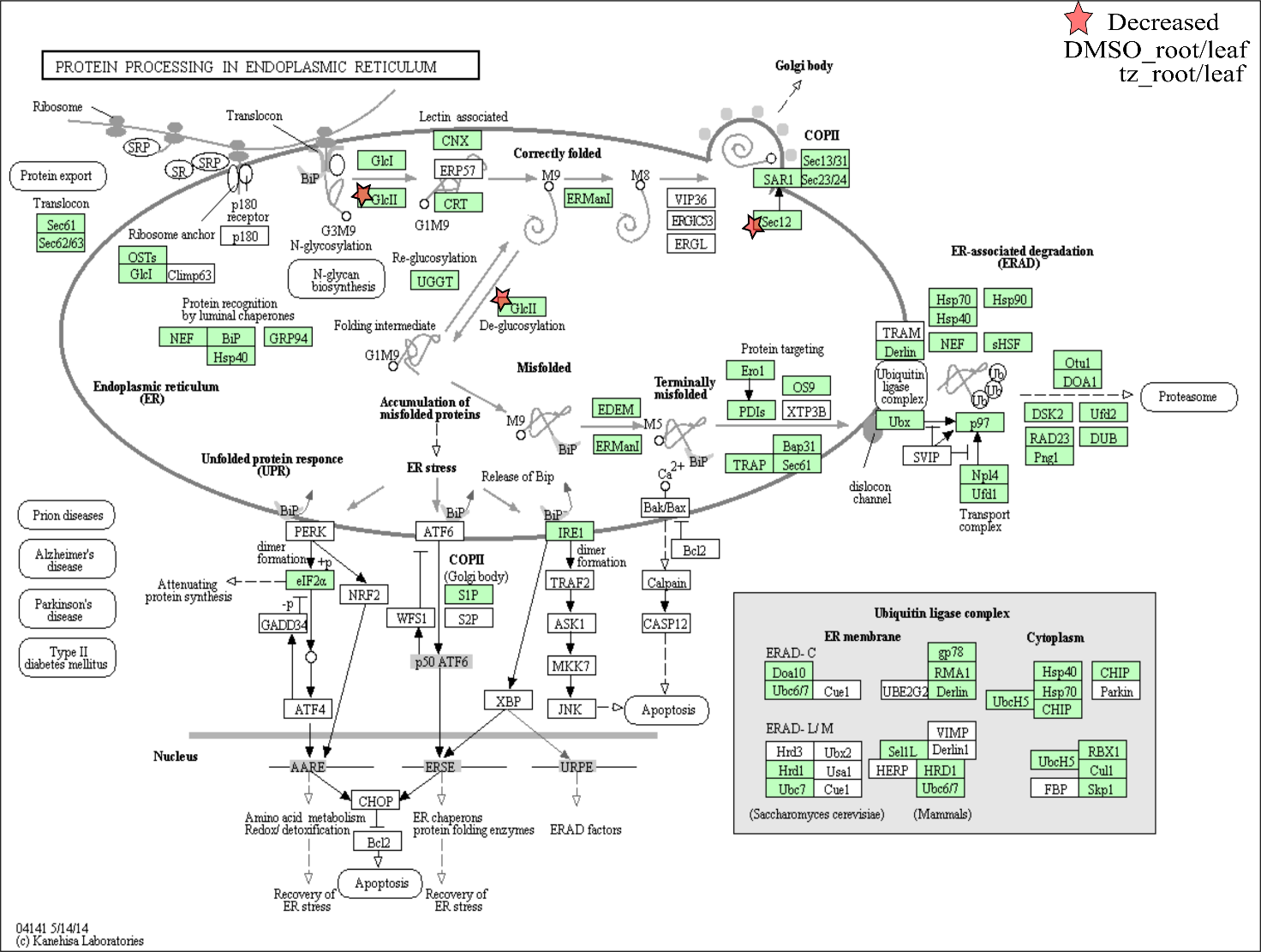

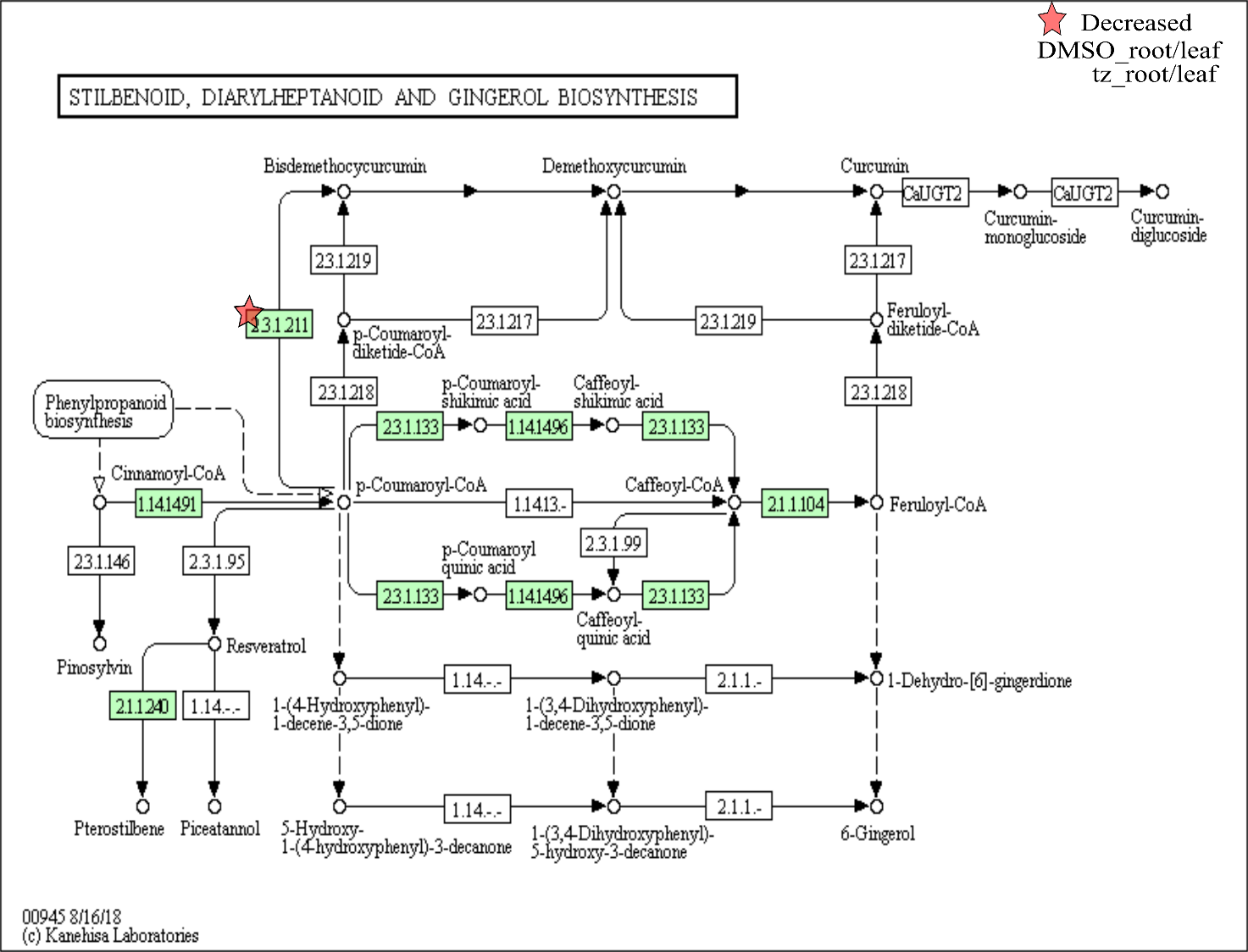


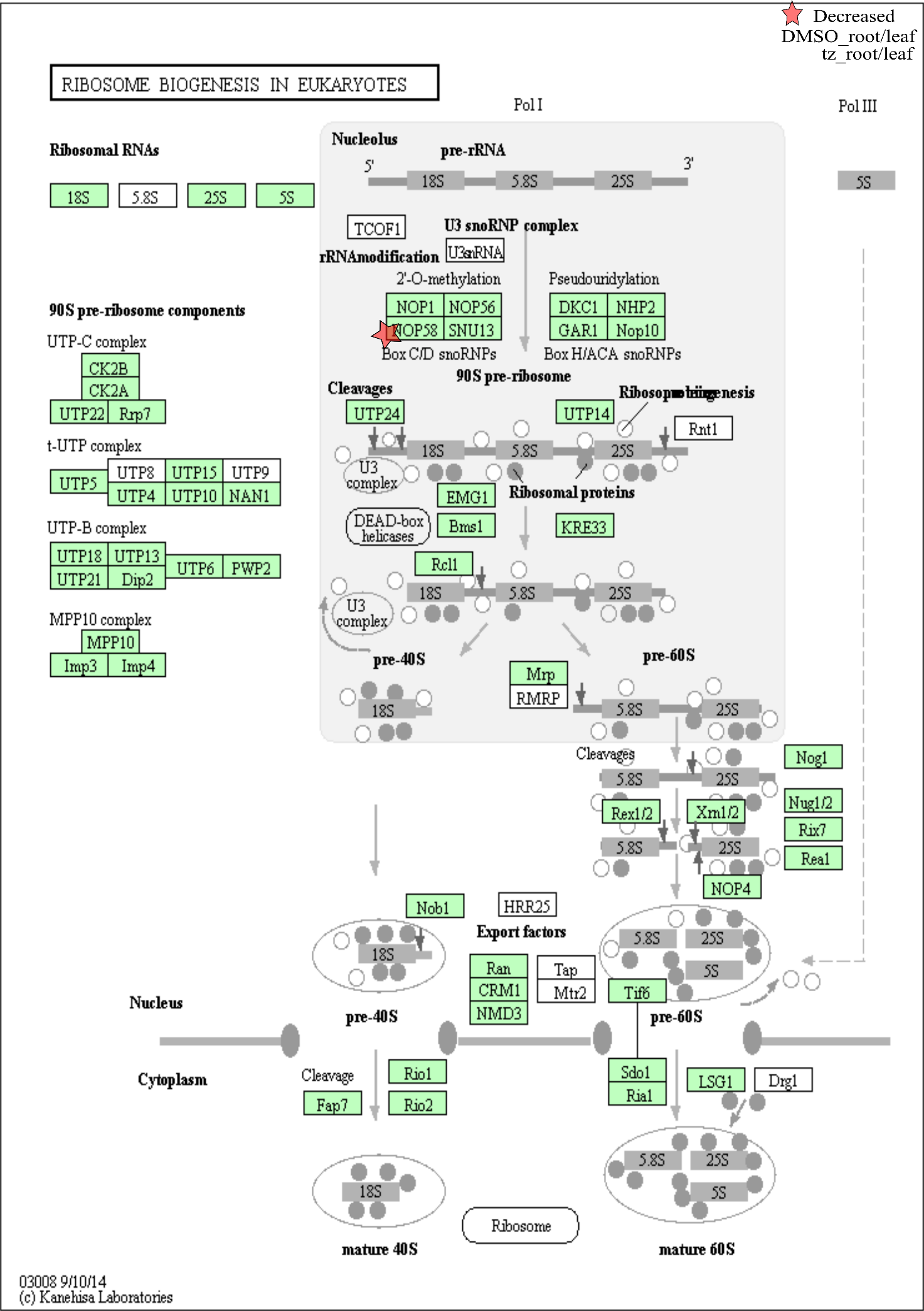


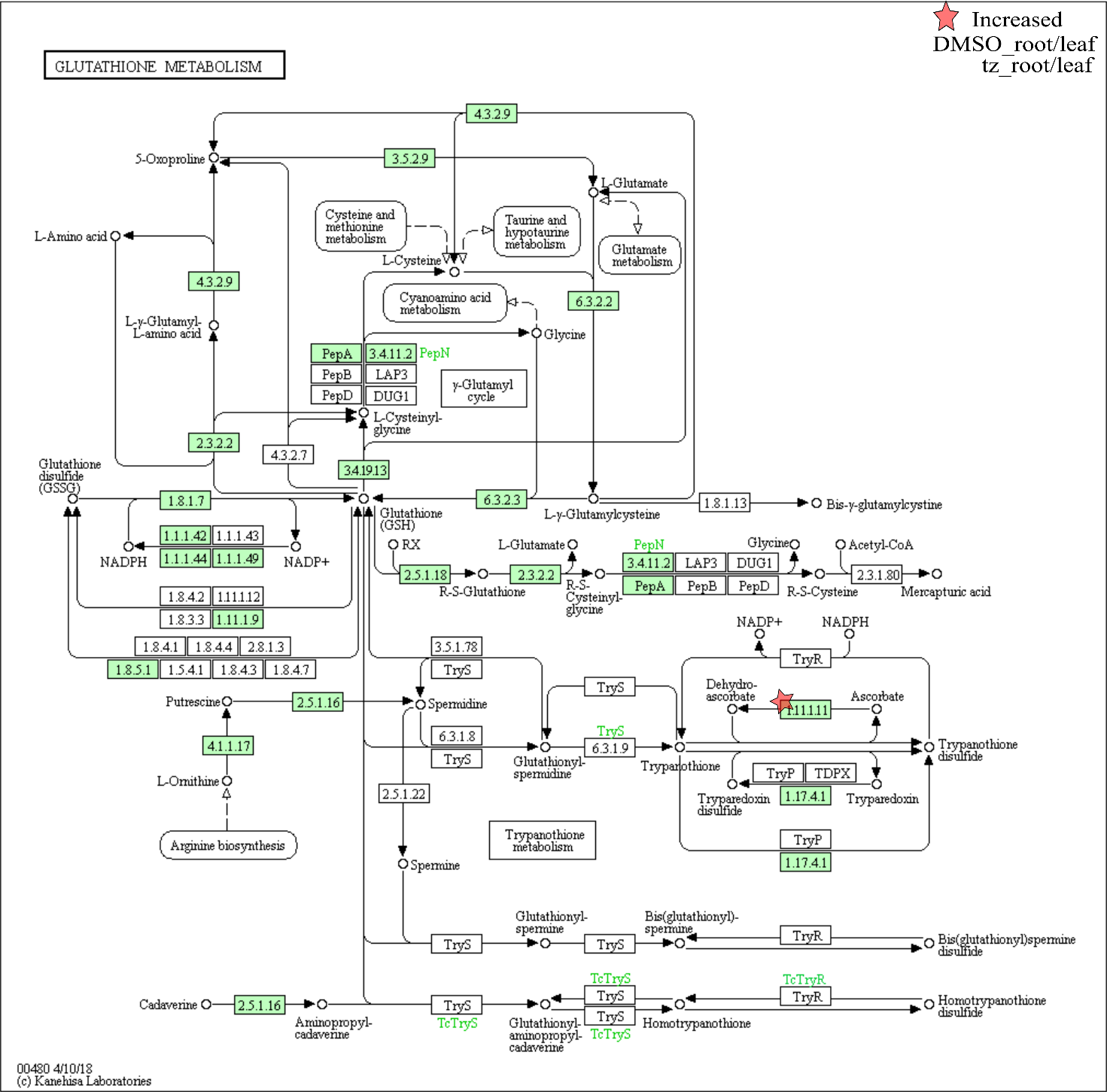


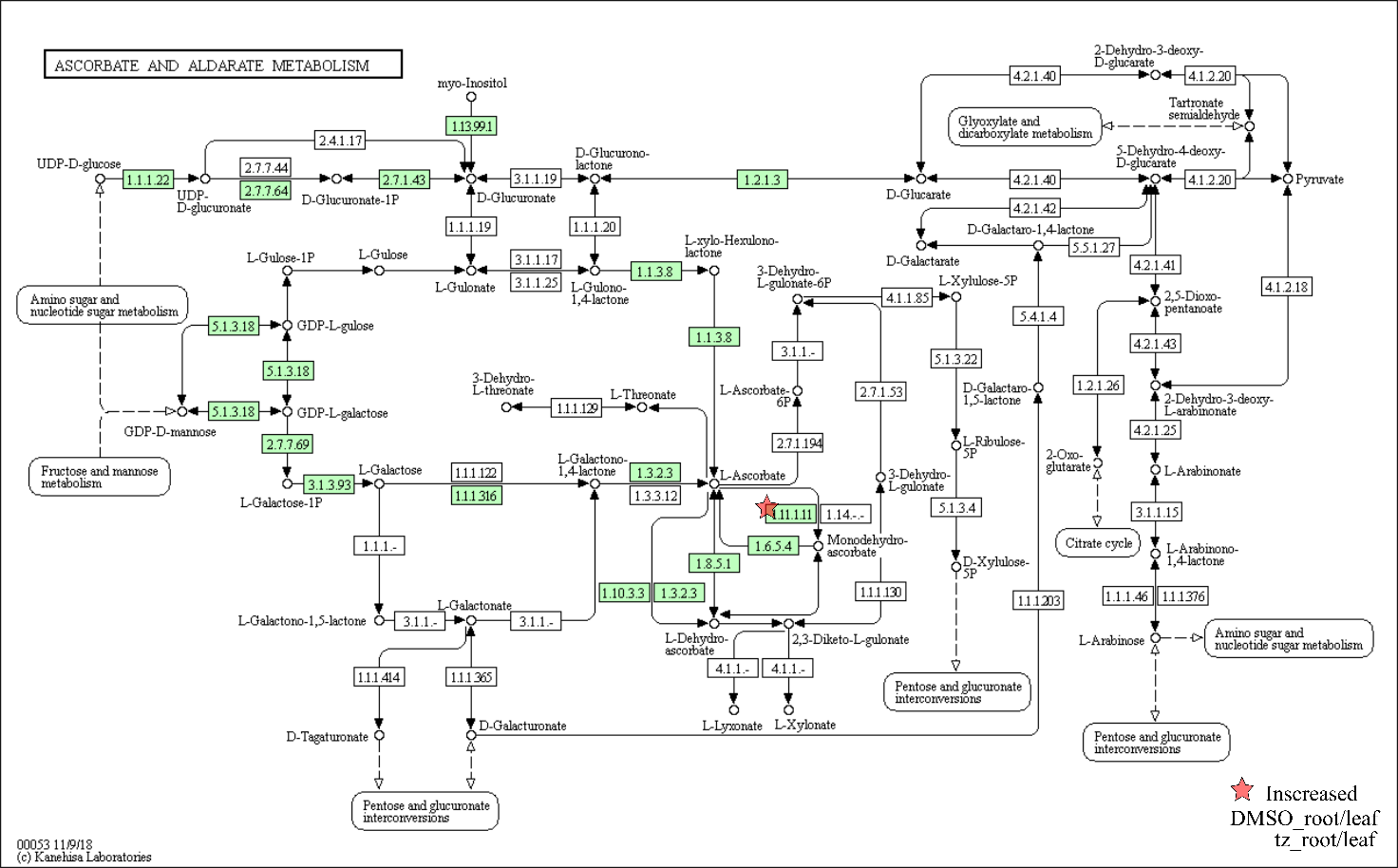


Figure: S1_fig: KEGG pathway map analyses of differentially expressed genes with treatment DMSO and trans-zeatin (tz) (a) Protein processing in endoplasmic reticulum, (b) Stilbenoid, diarylheptanoid and gingerol biosynthesis, (c) Ascorbate and aldarate metabolism, (d) Glutathione metabolism, (e) Ribosome biogenesis in eukaryotes
