# Supplementary material for: Identification of Potential Cytokinin Responsive Key Genes in Rice Treated With Trans-Zeatin Through Systems Biology Approach
Source: Front Genet. 2022 Feb 7;12:780599. doi: 10.3389/fgene.2021.780599 (PMC8859635; doi:10.3389/fgene.2021.780599)
Supplement: Supplementary file 3 [file DataSheet1.docx]

Table 1: Identified hub genes with respect to their level of connectivity in Turquoise module

| **S.No.** | **Hub Gene** | **Function/Annotation** | **Locus location** | **Acc. No.** |
| --- | --- | --- | --- | --- |
| 1 | Os.23857.1.S1_at | Horcolin/Jasmonate-induced protein, putative, expressed | LOC4335407/Chromosome 4, NC_029259.1 (12989808..12993215) | AK067477 |
| 2 | Os.7405.1.S1_at | uncharacterized LOC4333157 | LOC4333157/Chromosome 3, NC_029258.1 (17780383..17784105) | AK100427 |
| 3 | Os.26399.1.S1_at | MADS-box transcription factor 57 | LOC4330621/Chromosome 2, NC_029257.1 (30456659..30462758) | AY177702 |
| 4 | Os.38090.1.S1_at | uncharacterized LOC4324676 | LOC4324676/Chromosome 1, NC_029256.1 (21151568..21155639, complement) | AK068120 |

Table 2: Identified hub genes with respect to their level of connectivity in Blue module

| **S.No.** | **Hub Gene** | **Function** | **Locus location** | **Acc. No.** |
| --- | --- | --- | --- | --- |
| 1 | Os.10178.1.S1_a_at | probable cellulose synthase A catalytic subunit 3 [UDP-forming] | LOC4343049/Chromosome 7, NC_029262.1 (13741571..13747205, complement) | AK120236 |
| 2 | Os.52903.1.S1_at | uncharacterized LOC4329457 | LOC4329457/Chromosome 2, NC_029257.1 (18343563..18347153, complement) | AK069817 |
| 3 | Os.49559.1.S1_at | uncharacterized LOC4345975 | LOC4345975/Chromosome 8, NC_029263.1 (24986078..24990484) | AK067190 |
| 4 | Os.6899.2.S1_x_at | glucosidase 2 subunit beta | LOC4324264/Chromosome 1, NC_029256.1 (9702711..9709700, complement) | AK108476 |

Table 3: Identified hub genes with respect to their level of connectivity in Brown module

| **S.No.** | **Hub Gene** | **Function** | **Locus location** | **Acc. No.** |
| --- | --- | --- | --- | --- |
| 1 | Os.32537.1.S1_s_at | ATP-dependent protease La domain containing protein, expressed | LOC4326165/Chromosome 1, NC_029256.1 (31078612..31088072, complement) | AK102317 |
| 2 | Os.2106.1.S2_at | Os01g0512300 | Os01g0512300/Chromosome 1, NC_008394.3 (18047906..18050788, complement) | AK107048 |
| 3 | Os.12381.1.S1_x_at | dirigent protein 5 | LOC4344033/Chromosome 7, NC_029262.1 (26445635..26446517, complement) | AK106022 |
| 4 | Os.23808.1.S1_x_at | uncharacterized LOC4337691 | LOC4337691/Chromosome 5, NC_029260.1 (1652011..1653860) | AK108556 |

Table 4: Identified hub genes with respect to their level of connectivity in Green module

| **S.No.** | **Hub Gene** | **Function** | **Locus location** | **Acc. No.** |
| --- | --- | --- | --- | --- |
| 1 | Os.20555.1.S1_at | uncharacterized LOC4342515 | LOC4342515/Chromosome 7, NC_029262.1 (3773297..3779772) | AK100663 |
| 2 | Os.11903.1.S1_at | serpin-ZXB | LOC4333434/Chromosome 3, NC_029258.1 (23054420..23068346) | AK107194 |
| 3 | Os.14101.1.S1_at | exocyst complex component EXO70B1 | LOC4327525/Chromosome 1, NC_029256.1 (35412698..35416714) | AK122173 |
| 4 | Os.15367.1.S1_at | KH domain-containing protein At4g18375 | LOC4344900/Chromosome 8, NC_029263.1 (5810049..5816102, complement) | AK067859 |

Table 5: Identified hub genes with respect to their level of connectivity in Yellow module

| **S.No.** | **Hub Gene** | **Function** | **Locus location** | **Acc. No.** |
| --- | --- | --- | --- | --- |
| 1 | Os.54874.1.S1_at | protein GAMETE EXPRESSED 1 | LOC4347181/Chromosome 9, NC_029264.1 (16443693..16448158) | AK106970 |
| 2 | Os.17125.1.S1_at | putative L-ascorbate peroxidase 6 | LOC4346078/Chromosome 8, NC_029263.1 (25971775..25974968, complement) | AK065893 |
| 3 | Os.17490.1.A1_at | chlorophyll(ide) b reductase NOL, chloroplastic | LOC4333604/Chromosome 3, NC_029258.1 (25520290..25525342, complement) | CB669633 |
| 4 | Os.30000.1.S1_at | benzyl alcohol O-benzoyltransferase | LOC4343545/Chromosome 7, NC_029262.1 (21854513..21856895) | AK109553 |

Table 6: Identified hub genes with respect to their level of connectivity in Red module

| **S.No.** | **Hub Gene** | **Function** | **Locus location** | **Acc. No.** |
| --- | --- | --- | --- | --- |
| 1 | Os.18879.1.S1_at | boron transporter 1 | LOC4352546/Chromosome 12, NC_029267.1 (23248819..23253256) | AK100510 |
| 2 | Os.52480.1.S1_at | ribulose bisphosphate carboxylase/oxygenase activase, chloroplastic | LOC4337267/Chromosome 4, NC_029259.1 (33575149..33579656, complement) | AK067399 |
| 3 | Os.9121.1.S1_at | DNA primase small subunit | LOC9267485/Chromosome 5, NC_029260.1 (17006498..17011733, complement) | AK073973 |
| 4 | Os.11573.2.A1_a_at | ribulose bisphosphate carboxylase/oxygenase activase, chloroplastic | LOC4351224/Chromosome 11, NC_029266.1 (28932976..28936094, complement) | CB673145 |

Table 7: Identified hub genes with respect to their level of connectivity in Black module

| **S.No.** | **Hub Gene** | **Function** | **Locus location** | **Acc. No.** |
| --- | --- | --- | --- | --- |
| 1 | Os.19165.1.S1_at | uncharacterized LOC4350546 | LOC4350546/Chromosome 11, NC_029266.1 (17379762..17382920) | AK068341 |
| 2 | Os.56176.1.S1_at | bisdemethoxycurcumin synthase | LOC4342896/Chromosome 7, NC_029262.1 (10018732..10020733) | AK109558 |
| 3 | Os.17123.1.S1_at | uncharacterized LOC9271634 | LOC9271634/Chromosome 9, NC_029264.1 (18576964..18581257) | AK061852 |
| 4 | Os.39066.1.S1_at | lichenase-2 | LOC4338611/Chromosome 5, NC_029260.1 (18106236..18110996, complement) | CB628871 |

Table 8: Identified hub genes with respect to their level of connectivity in Pink module

| **S.No.** | **Hub Gene** | **Function** | **Locus location** | **Acc. No.** |
| --- | --- | --- | --- | --- |
| 1 | Os.19410.1.S1_at | SEC12-like protein 1 | LOC4342601/Chromosome 7, NC_029262.1 (4682485..4687647) | AK111777 |
| 2 | Os.50975.1.S1_at | bradykinin-potentiating and C-type natriuretic peptides | LOC4339650/Chromosome 5, NC_029260.1 (28310927..28311938) | AK059883 |
| 3 | Os.50361.2.S1_x_at | aspartic proteinase-like protein 2 | LOC4335504/Chromosome 4, NC_029259.1 (15623634..15653865, complement) | AK120870 |
| 4 | Os.49124.1.S1_at | uncharacterized LOC4332880 | LOC4332880/Chromosome 3, NC_029258.1 (13927427..13935150, complement) | AK064512 |

Table 9: Identified hub genes with respect to their level of connectivity in Grey module

| **S.No.** | **Hub Gene** | **Function** | **Locus location** | **Acc. No.** |
| --- | --- | --- | --- | --- |
| 1 | Os.35257.1.S1_at | hexose carrier protein HEX6 | LOC4342334/Chromosome 7, NC_029262.1 (1669273..1671384) | AK068296 |
| 2 | Os.11854.1.S1_at | probable plastid-lipid-associated protein 4, chloroplastic | LOC4333849/Chromosome 3, NC_029258.1 (28304914..28307925) | AK070474 |
| 3 | Os.16220.1.A1_at | probable aminotransferase ACS12 | LOC4340002/Chromosome 6, NC_029261.1 (1629690..1633597) | AK065212 |
| 4 | Os.10655.1.S1_at | 14 kDa zinc-binding protein | LOC4332685/Chromosome 3, NC_029258.1 (11672780..11677395) | AK121029 |
